# Prevalence of rectal carbapenem resistant *Enterobacterales* carriage among patients attending healthcare facilities in Ibadan, Nigeria: a descriptive study

**DOI:** 10.1186/s12879-024-09627-z

**Published:** 2024-07-24

**Authors:** Olukemi Adekanmbi, Oluwafemi Popoola, Adeola Fowotade, Olusola Idowu, Babatunde Ogunbosi, Sulaiman Lakoh, Ini Adebiyi, Omobolaji Ayandipo, Ayodele Olukayode Iyun

**Affiliations:** 1 Department of Medicine, College of Medicine, University of Ibadan, Queen Elizabeth Road, Mokola, Ibadan Nigeria; 2 Department of Community Medicine, College of Medicine, University of Ibadan, Queen Elizabeth Road, Mokola, Ibadan Nigeria; 3 Department of Medical Microbiology and Parasitology, College of Medicine, University of Ibadan, Queen Elizabeth Road, Mokola, Ibadan Nigeria; 4 Department of Anaesthesia, College of Medicine, University of Ibadan, Queen Elizabeth Road, Mokola, Ibadan Nigeria; 5 Department of Paediatrics, College of Medicine, University of Ibadan, Queen Elizabeth Road, Mokola, Ibadan Nigeria; 6 Department of Medicine, College of Medicine and Allied Sciences, University of Sierra Leone, Freetown, Sierra Leone; 7 Department of Medical Microbiology and Parasitology, University College Hospital, Queen Elizabeth Road, Mokola, Ibadan Nigeria; 8 Department of Surgery, College of Medicine, University of Ibadan, University College Hospital, Queen Elizabeth Road, Mokola, Ibadan Nigeria; 9 Department of Plastic, Reconstructive and Aesthetic Surgery, University College Hospital, Queen Elizabeth Road, Mokola, Ibadan Nigeria

**Keywords:** Carbapenem resistant *enterobacterales*, Antimicrobial resistance, Antimicrobial stewardship, Infection prevention and control, Bacteria

## Abstract

**Background:**

Carbapenem Resistant *Enterobacterales* (CRE) infections are increasingly associated with or directly responsible for morbidity and mortality from bacterial infections in sub-Saharan Africa where there are limited antibiotic options. CRE rectal colonization of patients in healthcare facilities provides a reservoir of these organisms and could potentially cause invasive infections in these settings. The prevalence of rectal carriage among patients attending healthcare facilities in Nigeria has not been previously described. We set out to assess the prevalence of rectal CRE carriage and their antibiotic susceptibility patterns among patients attending healthcare facilities in Nigeria.

**Methods:**

A descriptive cross-sectional study was carried out from December 2021 to September 2022 in Ibadan, in which patients attending primary, secondary and tertiary healthcare facilities were screened for rectal carriage of CRE by microscopy, culture and sensitivity of rectal swab specimens.

**Results:**

A total of 291 patients were screened; 45 (15.5%), 66 (22.7%) and 180 (61.8%) at primary, secondary and tertiary healthcare facilities, respectively. All but one of them had received a third-generation cephalosporin or carbapenem in the preceding 30 days. The mean age was 28.8 years and 55.7% were male. Overall, 51 (17.5%) participants had CRE colonization, with 5(11.1%), 9(13.6%) and 37(20.6%) at primary, secondary and tertiary healthcare facilities, respectively (*p* = 0.243). Regarding antimicrobial susceptibility, 43(84.3%) CRE isolates were resistant to at least 3 different classes of antibiotics while two *Escherichia coli* isolates were resistant to all 5 classes of antibiotics tested. The lowest rates of CRE resistance were to tigecycline (6, 11.5%) and colistin (8, 15.7%).

**Conclusions:**

In this first study on CRE colonization in Nigeria, we found that a substantial proportion of patients in three levels of healthcare facilities had rectal carriage of CRE, including pan-resistant isolates. Active surveillance and appropriate infection prevention and control practices (IPC) need to be urgently strengthened to mitigate the risk of active CRE infection.

**Trial registration:**

Not applicable.

**Supplementary Information:**

The online version contains supplementary material available at 10.1186/s12879-024-09627-z.

## Background

As a group, *Enterobacterales* are the leading cause of healthcare associated and community acquired infections [1]. Carbapenem resistant *Enterobacterales* (CRE) are resistant to carbapenems, often the last line of antibiotics available to treat multi-drug resistant gram-negative infections in resource limited settings [2]. Using predictive statistical models, the Global Burden of antimicrobial resistance (AMR) 2019 study estimated the all-age mortality rates attributable to AMR to be highest in the West African sub-region [3]. In addition, the commonly encountered *Enterobacterales*, *Escherichia coli* and *Klebsiella pneumoniae* were found to be the two leading pathogens responsible for death associated with AMR in 2019 [3]. The human gut serves as a reservoir for *Enterobacterales* and can be a source for invasive *Enterobacterales* infections to the host itself as well as those around them. There is evidence to support the role of colonizing *Enterobacterales* leading to invasive infection at other anatomical sites within the individual [4, 5]. Studies have shown that a significant proportion of patients with colonization can progress to invasive infection in a matter of days [over one-third in one intensive care unit (ICU) cohort, within a mean period of 6.5 days] [6]. In addition, globalization and rapid travel between countries and regions has made CRE colonization and infection data important globally. A study in the Netherlands found recent hospitalization abroad to be a risk factor for colonization with CRE [7]. A study from China showed that certain factors such as being hospitalized more than three times, being in a coma and receiving carbapenems are risk factors for invasive CRE disease in patients with previous colonization [8].

All of these suggest that exploring the burden of CRE colonization in any patient population might provide a signal for the potential burden and risk of invasive CRE disease in that population. CRE were known to be associated with healthcare associated infections and not community acquired infections. However, given the widespread and often unregulated use of antibiotics in low-and-middle-income countries (LMICs), increase in complex outpatient medical procedures and other frequent exposures to healthcare, it is not surprising that CRE colonization may be seen in patients without the traditional risk factors and that CRE infections are increasingly community acquired.

There are limited data about colorectal carriage of CRE in patients attending healthcare facilities in sub-Saharan Africa in general and Nigeria in particular [9]. While there exist data on invasive clinical infections including molecular characteristics, there are no published studies on fecal carriage of CRE in patients at primary, secondary and tertiary healthcare hospitals in Nigeria and rectal screening is not carried out routinely in Nigerian hospitals. There are policies aimed at preventing and controlling CRE infections at hospitals in Nigeria. These are mainly related to hand hygiene and regulation of broad spectrum antibiotic use. Implementation of these policies is inconsistent because of inadequate resources to support antimicrobial stewardship (AMS) and IPC.

Screening for CRE carriage is a preemptive and integral component of a multimodal approach to preventing invasive CRE infections along with other strategies such as handwashing and staff education [9, 10]. To fill a significant data gap regarding CRE colonization in Nigeria, we set out to measure the prevalence of CRE rectal colonization in select patients attending healthcare facilities in Ibadan, Nigeria. We also studied the antimicrobial susceptibilities of the CRE isolates.

## Methods

### Study setting and sites

The study was designed to take place concurrently in 3 West-African countries namely, Nigeria, Sierra Leone and Ghana. This is a report of the research at the Nigerian sites as the study has not yet been completed at the other sites. The study took place in Ibadan, Nigeria’s third most populous city with a population of 3.9 million, located in Southwestern part of the country [11]. It was a cross-sectional study carried out at one tertiary hospital, 3 secondary hospitals and 3 primary healthcare centers (PHCs). The main teaching hospital in Ibadan, the University College Hospital (UCH) was selected along with 3 secondary facilities: (Oni Memorial Children’s Hospital, Ring Road State Hospital and Our Lady of Apostles (OLA) Catholic Hospital, Oluyoro) and 3 PHCs. The PHCs selected were ALGON, Oke Adu and Oniyanrin PHCs, they are all located outside the local government areas (LGAs) in which the secondary and tertiary facilities are located.

The UCH is situated in Ibadan North Local Government Area (LGA) and its immediate catchment covers all 5 urban LGAs of Ibadan city with an estimated population of about 3 million individuals. The Ring Road State Hospital is a 200 - bed hospital which provides a comprehensive suite of medical and surgical services, drawing patients from the entire city of Ibadan and surrounding communities. It is a referral center for other primary and secondary facilities in the city and refers necessary cases out to the UCH. The Oni Memorial Children’s Hospital is a 62 bed state owned secondary healthcare facility which serves the city of Ibadan. OLA catholic hospital is a mission hospital with 160 beds which provides medical, surgical, paediatric, obstetric and gynaecologic services to patients from all over the city of Ibadan. The PHCs largely serve the communities in which they are located.

### Sampling method

The sample size was calculated using WinPepi version 11.65 [12]. The sample was determined to estimate a CRE rate of 100 per 1000 with an acceptable difference of 20 per 1000 at 95% confidence. This derived a minimum sample of 865 across three countries. We aimed to recruit a total of 900 (300 per country); 60%, 25% and 15% of patients to be recruited from tertiary, secondary and primary healthcare facilities respectively. A total of 291 patients were eventually recruited in Nigeria as follows: 180 at the tertiary hospital, 66 at the secondary facilities and 45 at the primary healthcare facilities. The target of 75 patients at the secondary level fell short by 7 patients because of low hospital utilization and slow recruitment rates till the end of the study period.

Certain criteria were used to identify patients at high risk of CRE colonization at primary, secondary and tertiary healthcare facilities. The criteria included exposure to carbapenems or 3rd or 4th generation cephalosporins in the preceding 30 days, ICU care, burn unit admission, hemodialysis, abdominal surgery and bladder catheterization for more than 48 h. See full list of enrollment criteria in additional file 1.

Patients with active lower gastrointestinal bleed, rectal or peri-rectal pathology and diagnosed invasive CRE infection were excluded from the study. This was to avoid causing discomfort to the patient, provoking a bleed or exacerbating the condition.

### Clinical and Laboratory methods

Informed consent was obtained from patients who met inclusion criteria. Rectal swabs were collected by trained research nurses and transported in a cold box to the on-site microbiology laboratory at the tertiary hospital for processing. The temperature of the cooler box was maintained at between 2 and 8 degrees centigrade. Pre-conditioned gel packs and temperature data loggers were placed inside the cooler boxes. Samples were placed in a manner that allows even cooling. During transit, continuous monitoring of the temperature was done using the data logger. Upon arrival, the temperature records were checked to ensure the samples were kept within the desired range. Specimens collected from the primary and secondary centers were transported to the laboratory at the tertiary center in Amies transport medium. Patients were recruited from medical, surgical and pediatric wards after admission. At the primary healthcare centers, they were recruited during their ambulatory visit to the center.

**Fig. 1:**
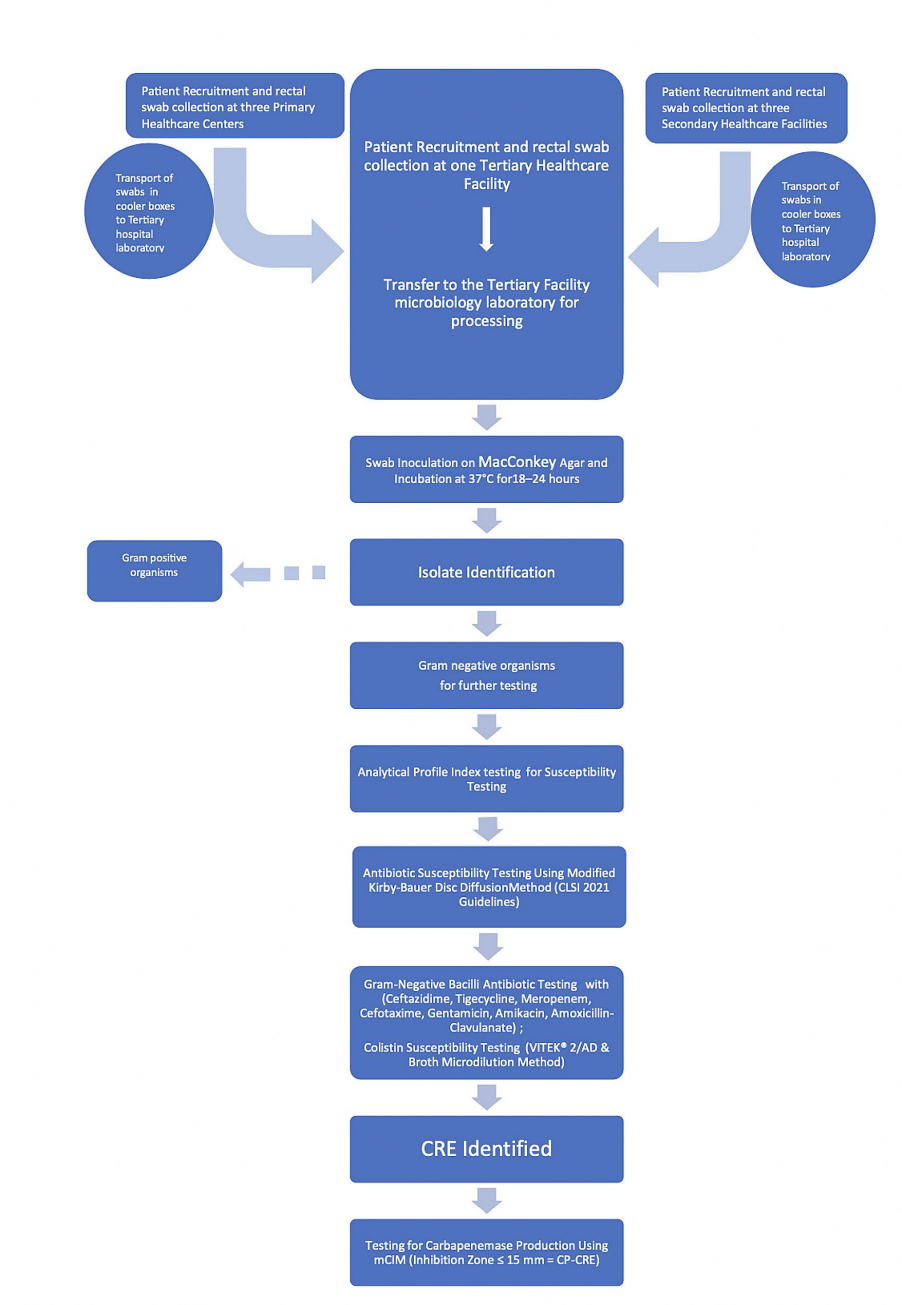
Flowchart of Clinical and Laboratory Methods

Swabs were inoculated onto MacConkey agar and incubated aerobically (at 37⁰C) for 18–24 h. Isolates were Gram stained and identified using catalase and coagulase reactions (Gram-positive organisms) and Analytical Profile Index (API) kit (Biomerieux, France) (Gram-negative bacilli). Antibiotic susceptibility was done using the Modified Kirby Bauer disc diffusion method and the results were expressed as susceptible, intermediate, or resistant according to the 2021 Clinical and Laboratory Standards Institute (CLSI) guidelines [13]. Briefly, all Gram-negative bacilli were tested using ceftazidime (30 µg), tigecycline(15 µg), meropenem(10 µg), colistin, cefotaxime(10 µg), gentamicin(10 µg), amikacin(30 µg), and amoxicillin-clavulanate (20/10µg)) antibiotic discs. Colistin susceptibility was determined by VITEK^®^ 2/AD and compared with the reference standard broth microdilution (BMD) according to the ISO 20776-1:2007 and 2021 CLSI guidelines [13] (Fig. 1).

Intermediate susceptibility and resistance to carbapenems were tested using modified Kirby Bauer disc diffusion method and evaluated according to the CLSI-2021 guidelines. For the purposes of analysis and discussion all intermediate and resistant isolates were classified as CRE. *Escherichia coli* (ATCC 25,922), *Staphylococcus aureus* (ATCC 25,923) and *Pseudomonas aeruginosa* (ATCC 27,853) control strains were used as control strains. As described by CLSI, carbapenemase production was tested using the modified carbapenamase inhibition method (mCIM). The mCIM is a phenotypic test to detect carbapenemase production in bacteria, particularly *Enterobacterales*. In this method, a carbapenem disk (e.g., meropenem) is incubated with a suspension of the test organism, then placed on a Mueller-Hinton agar plate inoculated with *Escherichia coli* (ATCC 25,922) and incubated overnight. Carbapenemase producing bacteria inactivate the carbapenem, resulting in little to no inhibition zone around the disk, allowing *E. coli* to grow close to the disk. In contrast, a clear inhibition zone indicates no carbapenemase production. An inhibition zone diameter of ≤ 15 mm was indicative of carbapenemase-producing carbapenem-resistant *Enterobacterales* (CP-CRE). E. coli ATCC 25,922 was used as quality control strain in antimicrobial susceptibility testing. E. coli NCTC-13,476 (blaIMP positive) and *K. pneumoniae* NCTC-13,440 (blaVIM-1 positive) were also used for positive control for mCIM.

Isolates were considered pan-resistant if they are resistant to at least one drug in all 5 classes of antibiotics or multi-drug resistant (MDR) if they are resistant to at least one antibiotic in three classes of antibiotics.

### Data analysis

Data were analyzed using the IBM SPSS package version 22. Categorical variables were summarized as proportions, normally distributed quantitative variables were summarized as means with standard deviation while skewed quantitative variables were summarized as medians with interquartile ranges. The proportion of patients with CRE were determined and compared across facilities using chi square tests. All statistical testing were conducted at α – 0.05.

## Results

### Demographic characteristics of study participants

Of the 291 study participants, 45 (15.5%), 66 (22.7%) and 180 (61.8%) were enrolled at primary, secondary and tertiary healthcare centers, respectively. There were 162 (55.7%) males and the mean age (SD) of all participants was 28.8 (24.9) years. The largest age group of patients were those aged 40–64 years, with 83 (28.5%) patients in that category (Table 1).

**Table 1:** Profile and selected care details of study participants

Characteristics	Carbapenem Resistant *Enterobacterales* colonization		Total Number (%)	
	Yes Number (%)	No Number (%)		Chi Sq *P* value
	51 (17.5)	240 (82.5)	291 (100)	
Gender				
Male	29 (17.9)	133 (82.1)	162 (55.7)	0.817
Female	22 (17.1)	107 (82.9)	129 (44.3)	
Age group				
Less than 1 year	7 (18.9)	30 (81.1)	37(12.7)	0.564
1–4 years	12 (25.0)	36 (75.0)	48 (16.5)	
5–14 years	4 (14.8)	23 (85.2)	27 (9.3)	
15–39 years	9 (12.7)	62 (87.3)	71 (24.4)	
40–64 years	16 (19.3)	67 (80.7)	83 (28.5)	
65 years and above	3 (12.0)	22 (88.0)	25 (8.6)	
Mean age (SD)	**27.1 (25.6)**	**29.3 (24.7)**	**28.9 (24.8)**	0.558
Level of care				
Primary	5 (11.1)	40 (89.9)	45 (15.5)	0.243
Secondary	9 (13.6)	57 (86.4)	66 (22.7)	
Tertiary	37 (20.6)	143 (79.4)	180 (61.9)	
ICU care				
Yes	2 (18.2)	9 (81.8)	11 (3.7)	0.848
No	49 (17.5)	231 (82.5)	280 (96.3)	
Admitting Specialty ward				
None (PHC/outpatient)	5 (11.1)	40 (88.9)	45(15.5)	0.0609
Medicine	14 (18.2)	63 (81.8)	77 (26.4)	
Paediatrics	23 (26.4)	64 (73.6)	87 (29.9)	
Surgery	7 (9.9)	64 (90.1)	71 (24.4)	
ICU/Burn	2 (18.2)	9 (81.8)	11 (3.8)	
*Enterobacterales*				
*Eschericia coli*	35 (19.2)	147 (80.8)	182 (72.8)	0.924
*Klebsiella spp.*	7 (25.9)	20 (74.1)	27 (10.8)	
*Enterobacter spp.*	3 (27.3)	8 (72.7)	11 (4.4)	
*Raoultella Ornitholytica*	2 (16.7)	10 (83.3)	12 (4.8)	
*Serratia Odorifera*	2 (18.2)	9 (81.8)	11 (4.4)	
Others	2 (28.6)	5 (71.4)	7 (2.8)	

### Colonization with CRE

Overall, 51 (17.5%) patients were colonized with CRE; 5 (11.1%) from primary facilities, 9 (13.6%) from secondary facilities and 37 (20.6%) from the tertiary facility. The type of ward with the highest prevalence of CRE colonization in patients enrolled in our study was the pediatric wards [23 (26.4%) of 87 patients sampled]. Of the 51 patients colonized with CRE, 5/51 (9.8%) were out-patients (all at PHCs), the majority, 23/51 (45.1%) were admitted to pediatric wards, 14/51 (27.5%) were admitted to adult medical wards while just 7/51 (13.7%) and 2/51 (3.9%) were admitted to surgical services and ICU/Burn wards respectively (percentages not shown in Table 1). The majority (35, 68.6%) of the CRE isolates were *E.coli*, followed by *Klebsiella* spp. (7, 13.7%) (Table 1).

### Risk factors and outcomes of CRE colonization

All but one of the 291 patients had either received a carbapenem or 3rd /4th generation cephalosporin within the 30 days prior to the participation in the study (Table 2). There were 104 (35.7%) patients with urinary catheters and 16 (31.4%) of them were colonized with CRE. On the other hand, only 35 (18.7%) of 187 patients without urinary catheters were colonized with CRE. The mean (SD) number of days on admission before participation in the study for those colonized and not colonized with CRE was 10.78(9.15) and 9.37(9.62) days, respectively. Outcomes of admission were known for 246 patients in the study; 17 of them died of whom only 2 (11.8%) were colonized with CRE.

**Table 2:** Clinical details of study participants

Characteristics	Carbapenem Resistant *Enterobacterales* colonization		Total Number (%)	
	Yes Number (%)	No Number (%)		Chi Sq *P* Value
Carbapenem or cephalosporin use				
Yes	51 (17.6)	239 (82.4)	290 (99.7)	1.000
No	0 (0)	1 (100.0)	1 (0.3)	
Abdominal surgery				
Yes	7 (14.3)	42 (85.7)	49 (16.8)	0.611
No	44 (18.2)	198 (81.8)	242 (83.2)	
Urethral catheter				
Yes	16 (31.4)	88 (68.6)	104 (35.7)	0.472
No	35 (18.7)	152 (81.3)	187 (64.3)	
Stroke admissions				
Yes	3 (15.4)	15 (84.6)	18 (6.2)	0.874
No	48 (17.9)	225 (82.1)	273 (93.8)	
Recent hospital admission				
Yes	7 (14.9)	40 (85.1)	47 (16.2)	0.605
No	44 (18.0)	200 (82.0)	244 (83.8)	
Mechanical ventilation				
Yes	1 (12.5)	7 (87.5)	8 (2.7)	0.816
No	50 (17.7)	233 (82.3)	283 (97.3)	
Hemodialysis				
Yes	1 (1.7)	13 (92.9)	14 (4.8)	0.378
No	50 (18.1)	227 (81.9)	277 (95.2)	
Days admitted before CRE test				
Mean (SD)	10.78 (9.15)	9.37 (9.62)	9.64 (9.53)	
Treatment outcome	*n* = 46	*n* = 200	*n* = 246	
Died	2 (11.8)	15 (88.2)	17 (6.9)	0.747
Discharged alive	44 (19.2)	185 (80.8)	229 (93.1)	

### Antibiotic resistance profile

Regarding sensitivity to other antibiotics, almost 100% of the CRE isolates were resistant to 3rd and 4th generation cephalosporins and amoxicillin-clavulanate (Table 3). Overall, there was 84.3% resistance to gentamicin and 71.9% resistance to amikacin. The lowest levels of resistance were to colistin (15.7%) and tigecycline (11.8%).

**Table 3:** CRE resistance to other antibiotics

Antibiotic	*n* (%) of resistant* Enterobacterales* isolates		
	***Klebsiella *** **spp .(n = 7)**	***E. coli*** **(n = 35)**	**All isolates (N = 51)**
Amoxicillin-clavulanate	7 (100)	32/34* (94.1)	47/49 (95.9)
Cefpodoxime	6 (85.7)	34 (97.1)	49 (96.1)
Cefotaxime	6 (85.7)	34 (97.1)	49 (96.1)
Ceftazidime	7 (100)	32 (91.4)	48 (94.1)
Gentamicin	4 (57.1)	30 (85.7)	43 (84.3)
Amikacin	3/6 (50.0)*	16/22 (72.7)*	23/32 (71.9)*
Levofloxacin	3/6 (50.0)*	30 (85.7)	40/50 (80)*
Colistin	1 (14.3)	6 (17.1)	8 (15.7)
Tigecycline	2 (28.6)	3 (8.6)	6 (11.8)

* Some isolates were not tested against stated antibiotic

Two *E. coli* isolates were pan-resistant (beta-lactams, aminoglycosides, quinolones, tigecycline and colistin), while none of the *Klebsiella spp.* were. Of all the 51 CRE isolates, 43 (84.3%) were MDR. Thirty (85.7%) and 5 (71.4%) of the *E.coli* and *Klebsiella* isolates respectively were MDR (Table 3) .

## Discussion

Our study evaluated colorectal carriage of *Enterobacterales* which are resistant to carbapenems in high-risk patients attending the 3 levels of healthcare facilities in Ibadan, Nigeria. A study which has hitherto not been conducted. We found carriage of CRE at all three levels of healthcare. Expectedly, the proportion of patients with CRE colonization increased from the PHC to tertiary level although this difference was not statistically significant (*P* = 0.2). This signifies that CRE carriage is not limited only to hospitalized patients previously considered to be ‘high-risk’ but also out-patients and possibly their healthy household contacts. It also reinforces the importance of (IPC) practices in healthcare facilities and water and sanitation in the community.

A study among hospitalized children in tertiary hospital In Lome, Togo revealed 6.9% fecal carriage of CRE [14]. Similar proportions of rectal carriage of CRE were reported among children admitted to an academic hospital in Cape Town [15]. These carriage rates are lower than the 17.5% of all recovered isolates and 26.4% of the patients admitted to pediatric wards found in our study.

Umar et al. found 14.9% CRE prevalence among cephalosporin resistant bacterial isolates obtained from clinical specimens at a tertiary hospital in Kano, Nigeria while Jamal and colleagues reported prevalences of 8% and 14% CRE among clinically significant *Enterobacterales* isolates in hospitalized patients in Nigeria and Kuwait, respectively [16, 17]. Suweiba et al. in Kaduna, Nigeria found 6.1% resistance of *E. coli* and 6.5% resistance of *Klebsiella spp*. to meropenem and ertapenem respectively among these isolates cultured from urine samples [18]. The latter three of these CRE studies involved isolates from clinical specimens (and not colorectal carriage), highlighting the paucity of data on screening for asymptomatic CRE carriage in Nigerian healthcare facilities. Given that only high-risk patients were screened in our study, we are unable to arrive at an estimate of the prevalence of CRE carriage in the general population of patients attending the healthcare facilities. However, it is notable that all but one of the patients in our study met the enrollment criterion of antibiotic use which is quite common in ours and similar facilities. A point prevalence study done at the University College Hospital in 2020 reported 59.6% of all patients surveyed on 38 different wards received at least one dose of antibiotics and the most frequently used antibiotic was a third-generation cephalosporin [19]. Of note, our study did not identify any significant risk factors for CRE colonization and that was probably due to the modest size of our study.

The importance of the possible downstream sequelae of CRE colonization cannot be over emphasized. A study in Oman showed that patients with CRE bacteremia who required ICU care were more likely to also be colonized with CRE [20]. Another study from China showed that CRE *K. pneumoniae* rectal colonization is an independent risk factor for CRE *K. pneumoniae* blood stream infection in allogeneic hematopoetic stem cell transplant patients [21]. Patients in ICUs have historically been targeted for CRE rectal surveillance in high income countries for multiple reasons including extensive antibiotic use and the critical health status of the patients which would make drug resistant infections all the more difficult to treat. Our study had relatively few ICU patients but nevertheless had substantial rates of CRE colonization.

We found that 11.1% of patients at the primary healthcare level were colonized with CRE. These were patients with antibiotic use as a predisposing factor rather than hospitalization and/or current in-patient healthcare exposure (all participants at the PHC level met the criterion of antibiotic use in the preceding 30 days). This finding was similar to a multi-center study in the US which showed 10% community acquired CRE colonization among hospitalized patients. [22]. This finding that over one in ten out-patients attending primary healthcare facilities in Ibadan who had received a cephalosporin antibiotic are colonized with CRE has great implications for potential spread of CRE within the community. We also found that in-patient pediatric patients also had a significantly higher proportion of CRE colonization than in-patient adult medical and surgical patients which might be related to high antibiotic use as previously described in our tertiary hospital [19]. Expectedly, there was almost universal resistance to 3rd generation cepahlosporins and amoxicillin-clavulanate. The lowest overall resistance in the CRE isolates was to tigecycline with a slightly higher overall resistance to colistin. This is similar to what was reported by Sader et al. from 70 US hospitals [23]. Notably, 2 of the 51 (3.9%) CRE isolates were resistant to all locally available antibiotics while 84.3% of them were multiple drug resistant.

Our study has some limitations. The high rates of CRE colonisation in our study population is likely higher than the prevalence in the populations accessing care in our healthcare facilities due to our selection criteria. Nevertheless, it demonstrates, for the first time in Nigeria, CRE carriage for patients attending all levels of healthcare. This calls for increased surveillance and evidence-based guidelines for targeted screening and appropriate IPC interventions where necessary. Given that only high-risk patients, rather than all patients attending the facilities were screened, we are not able to comprehensively assess for risk factors for CRE colonization among all patients attending these healthcare facilities. This needs to be investigated to better understand the burden of CRE colonisation in hospital and the community, and to guide IPC and water, sanitation and hygiene practices. This will help control spread of these MDR organisms and address attendant risk of progression to invasive CRE infection which is associated with high mortality rates and additional healthcare costs. In addition, our study did not evaluate molecular characteristics of the CRE isolates found. This would have provided information on the mechanisms of carbapenem resistance which would then inform the appropriate IPC practices to prevent the spread of such isolates. Lastly, our study did not evaluate the susceptibility of the CRE isolates to newer antibiotics for CRE such as ceftazidime-avibactam and cefidorecol. As such, we are unable to make specific recommendations for the expansion of access to specific antibiotics to treat invasive infections caused by these isolates.

## Conclusion

We have documented for the first time, a high magnitude of rectal colonization with CRE among high-risk patients attending primary, secondary and tertiary healthcare facilities in a major city in Nigeria. Patients in tertiary healthcare facilities and pediatric wards were more frequently colonized than other groups. IPC practices, antimicrobial stewardship and water sanitation and hygiene practices should be strengthened to prevent the spread of colonizing CRE in healthcare and community settings.

### Electronic supplementary material

Below is the link to the electronic supplementary material.

Supplementary Material 1

## Data Availability

All data supporting the findings of this study are available within the paper and its Supplementary information.

## Abbreviations

CRE: Carbapenem Resistant *Enterobacterales*
AMR: Antimicrobial Resistance
ICU: Intensive Care Unit
PHC: Primary Healthcare Center
UCH: University College Hospital
OLA: Our Lady of Apostles
LGA: Local Government Area
IPC: Infection Prevention and Control
CLSI: Clinical and Laboratory Standards Institute
EUCAST: European Committee on Antimicrobial Susceptibility Testing
