# Supplementary material for: Prevalence of rectal carbapenem resistant Enterobacterales carriage among patients attending healthcare facilities in Ibadan, Nigeria: a descriptive study
Source: BMC Infect Dis. 2024 Jul 24;24:726. doi: 10.1186/s12879-024-09627-z (PMC11267743; doi:10.1186/s12879-024-09627-z)
Supplement: Supplementary file 1 — Supplementary Material 1 [file 12879_2024_9627_MOESM1_ESM.docx]

**Supplementary Material**

*Criteria for CRE screening at tertiary centers:*

- Intensive care unit (ICU) patients with any exposure to 3^rd^ or 4^th^ generation cephalosporins or carbapenem in the prior 30 days
- ICU patients with any (including current) mechanical ventilation in the index hospitalization
- ICU patients transferred from another acute care facility where they were hospitalized for at least 48 hours
- Burn unit patients with any exposure to 3^rd^ or 4^th^ generation cephalosporins in the prior 30 days
- Burn unit patients transferred from another acute care facility where they stayed at least 48 hours
- In-born neonatal ward patients with any exposure to 3^rd^ or 4^th^ generation cephalosporins or carbapenem in the prior 30 days
- Out-born neonatal ward patients with any exposure to 3^rd^ or 4^th^ generation cephalosporins or carbapenem in the prior 30 days
- Patients on Medical or Surgical wards with exposure to any 3^rd^ or 4^th^ generation cephalosporin or carbapenem in the prior 30 days AND ***any*** of the following co-morbidities:

ESRD requiring hemodialysis

Gastrointestinal surgery

In-dwelling urethral or suprapubic catheter for > 48 hours

Stroke

*Criteria for CRE screening at Secondary centers:*

- Patient transferred from another acute care facility where were hospitalized for at least 48 hours
- Patients on Medical or Surgical wards with exposure to any 3^rd^ or 4^th^ generation cephalosporin or carbapenem in the prior 30 days AND **any** of the following co-morbidities:

Chronic kidney disease

Gastrointestinal surgery

In-dwelling urethral or suprapubic catheter for > 48 hours

Stroke

*Criteria for CRE screening at Primary centers:*

- Hospitalization > 48 hours at an acute care facility within the last 90 days
- Exposure to 3^rd^ or 4^th^ generation cephalosporins within the prior 30 days
- Exposure to carbapenems within the prior 30 days
- Outpatient indwelling urethral or suprapubic catheter
- On-going out-patient hemodialysis
